# Management of Temporomandibular Joint Reankylosis: A Case Series

**DOI:** 10.7759/cureus.39137

**Published:** 2023-05-17

**Authors:** Prasad Cheruvathur, Sethurajan Sethurajan Balasubramanian, Lavanya Lakshminarasimhan, Vasu Kumarandi

**Affiliations:** 1 Department of Oral and Maxillofacial Surgery, Tamil Nadu Government Dental College and Hospital, Chennai, IND; 2 Department of Anesthesiology and Critical Care, Tamil Nadu Government Dental College and Hospital, Chennai, IND

**Keywords:** ankylosis, esmarch, osteotomy, pesudojoint, trismus

## Abstract

Background

Temporomandibular joint ankylosis is a severe debilitating clinical condition where there is fusion of the mandible with the temporal bone. It is often a challenge to the maxillofacial surgeon as the surgical treatment protocol must be tailored individually according to the time of presentation of the ankylosis, and proper postoperative aggressive physiotherapy must be advocated, which is essential for a successful outcome. This is a case series of six recurrent temporomandibular joint ankylosis, in which the historical Esmarch surgery was done, and the pterygomassetric sling was interposed between the osteotomized segments. Postoperative mouth opening and surgical outcome were satisfactory. In our cases, we created a pseudojoint, which was very successful using the Esmarch procedure.

Aim

We aim to improve mouth opening in patients presenting with temporomandibular joint reankylosis using the Esmarch procedure and evaluate the efficacy of the conventional and modified Esmarch procedure.

Materials and methods

We have included six cases of recurrent temporomandibular joint reankylosis. Five cases were operated on using the conventional Esmarch procedure in which the osteotomy was done at the angle region, below the inferior alveolar nerve canal, and one case using the modified Esmarch procedure, wherein the osteotomy was done above the inferior alveolar nerve canal. The patients included in the case series presented with temporomandibular joint reankylosis and had undergone multiple surgeries for the release of ankylosis.

Results

Satisfactory postoperative mouth opening was achieved in all six patients. It was observed that in the modified Esmarch osteotomy, where the cuts were placed above the inferior alveolar nerve canal, there was a massive hemorrhage intraoperatively. This was primarily attributed to the altered anatomy of the maxillary artery, which was very close to the ankylotic mass. When the osteotomy was done below the inferior alveolar nerve canal, it was found that by this technique, the intraoperative hemorrhage was minimal, but it carries a risk of postoperative inferior alveolar nerve paresthesia, which was managed conservatively.

Conclusion

With the abovementioned results, we proceeded with the conventional Esmarch procedure for five cases and the modified Esmarch procedure for one case. It was found that in temporomandibular joint reankylosis cases, where there is extensive ankylotic mass extending from the glenoid fossa to the coronoid process of the mandible, this Esmarch procedure provides promising results when the osteotomy cuts are placed below the nerve canal.

## Introduction

Temporomandibular joint ankylosis is a clinical condition where the patient is unable to open the mouth due to the bony or fibrous union of the mandibular condyle with the glenoid fossa of the temporal bone. The etiology of it is varied and is mainly due to trauma or infection [1]. Clinical presentation varies at the time of involvement. During the growth phase, there is severe facial asymmetry due to stunted growth of the joint and the mandible along with associated facial musculature. In older individuals, it is more characterized by the prominent antegonial notch. Provisionally, the treatment of choice is the release of the joint surgically, mainly gap arthroplasty followed by either condyle reconstruction prosthesis or interposition flap.

The problem arises when repeated surgery is done due to reankylosis and massive callous formation, where the traditional method of gap arthroplasty is not feasible. In these conditions, the surgery of choice is to create a pseudojoint.

This article presents a case series of six patients who have undergone multiple surgeries due to reankylosis, resulting in massive callous formation involving the base of the skull. We have modified the surgical procedure following the Esmarch experience by creating a pseudojoint in the angle region [2].

## Materials and methods

Six patients with temporomandibular joint reankylosis were included in this case series. All the patients included in the case series presented with chief complaints of inability to open their mouths and gave a prior history of trauma to the jaws but did not seek any medical attention for the same. All the patients had undergone multiple surgeries for the release of ankylosis previously. Preoperatively, there was nil mouth opening in all six patients. The patients included belonged to the age group of 20-35 years and comprised five male and one female patient. Preoperative orthopantomogram and CT of facial bones were taken for all the patients. Conventional Esmarch angle osteotomy was done in five patients, and modified osteotomy was done in one patient. Each of the surgical procedures is described in detail.

Conventional Esmarch osteotomy

In this case, as described below, the patient had progressively declining mouth opening since the trauma and hence was operated surgically by gap arthroplasty on the left side with elective tracheostomy at the age of 11 years and bilaterally following reankylosis at the age of 15 years and 19 years. The patient had failed to undergo aggressive physiotherapy after every surgery and hence presented with nil mouth opening at the time of presentation to the outpatient department of Oral and Maxillofacial Surgery, Tamil Nadu Government Dental College and Hospital, Chennai.

**Figure 1:**
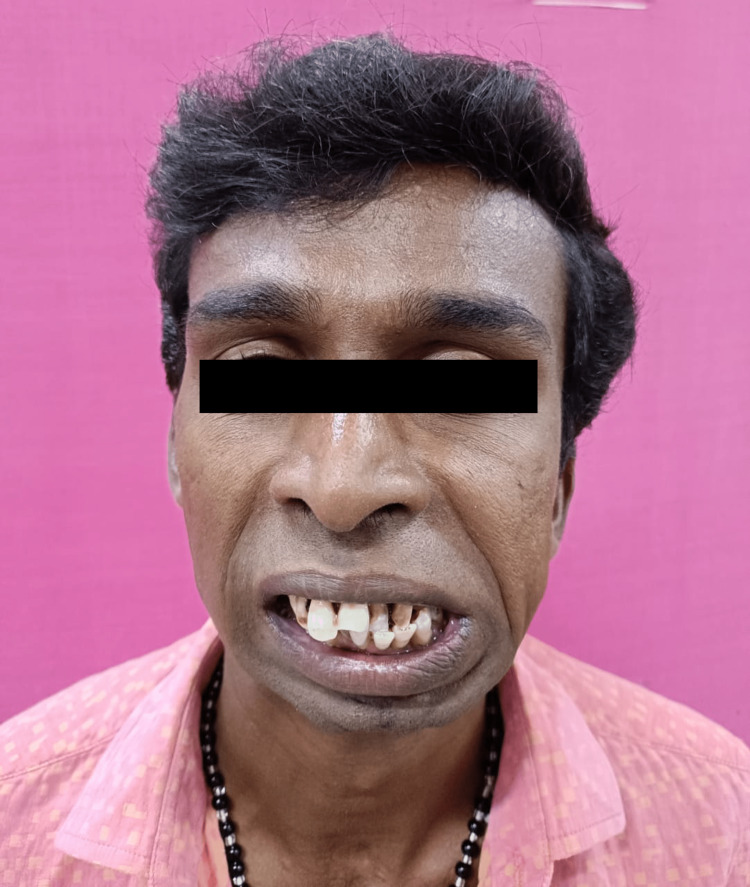
Preoperative nil mouth opening

Clinically, the patient presented with gross facial asymmetry, nil mouth opening, typical bird face appearance, posterior crossbite, and malocclusion (Figure 1).

**Figure 2:**
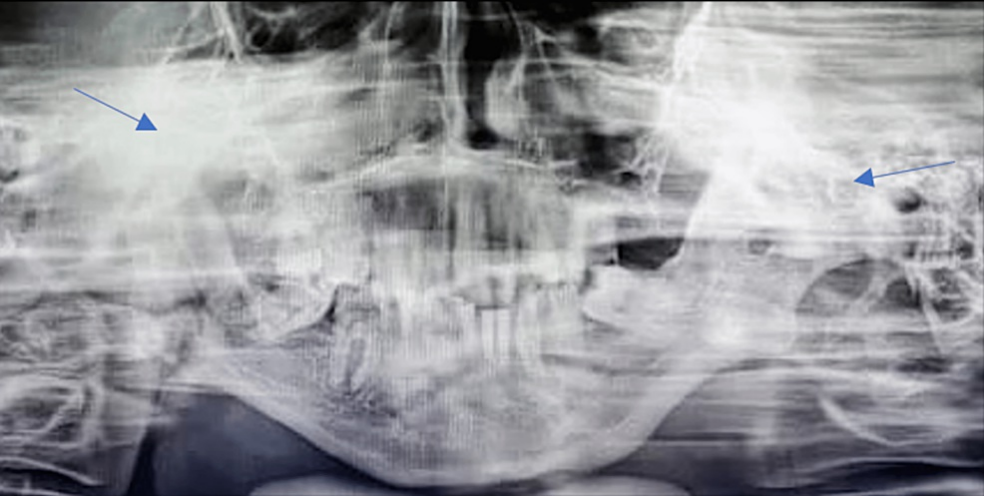
Preoperative orthopantomogram showing extensive ankylotic mass (arrows)

Radiographic investigations such as orthopantomogram and CT of the facial bones were taken, which revealed complete obliteration of the joint space, elongated coronoid process bilaterally, and complete bony fusion of the joint with extensive callous formation extending to the skull base (Figure 2). Virtual surgical planning along with a stereolithographic model was done preoperatively.

Surgical procedure

After a thorough evaluation, planning, and anesthetic evaluation, we decided to perform the conventional Esmarch osteotomy under general anesthesia. The majority of the ankylosis patients have an increased propensity for airway obstruction and obstructive sleep apnea mainly due to reduced pharyngeal airway space. In these cases, lateral and anteroposterior view neck X-ray was taken priorly to assess the airway space. Consent for elective tracheostomy was obtained in all cases as they had profound microgenia, which made chin lift and jaw thrust more difficult. The airway was preoperatively anesthetized with topical 10% xylocaine spray followed by awake fiberoptic nasotracheal intubation, thus eliminating the need for tracheostomy.

**Figure 3:**
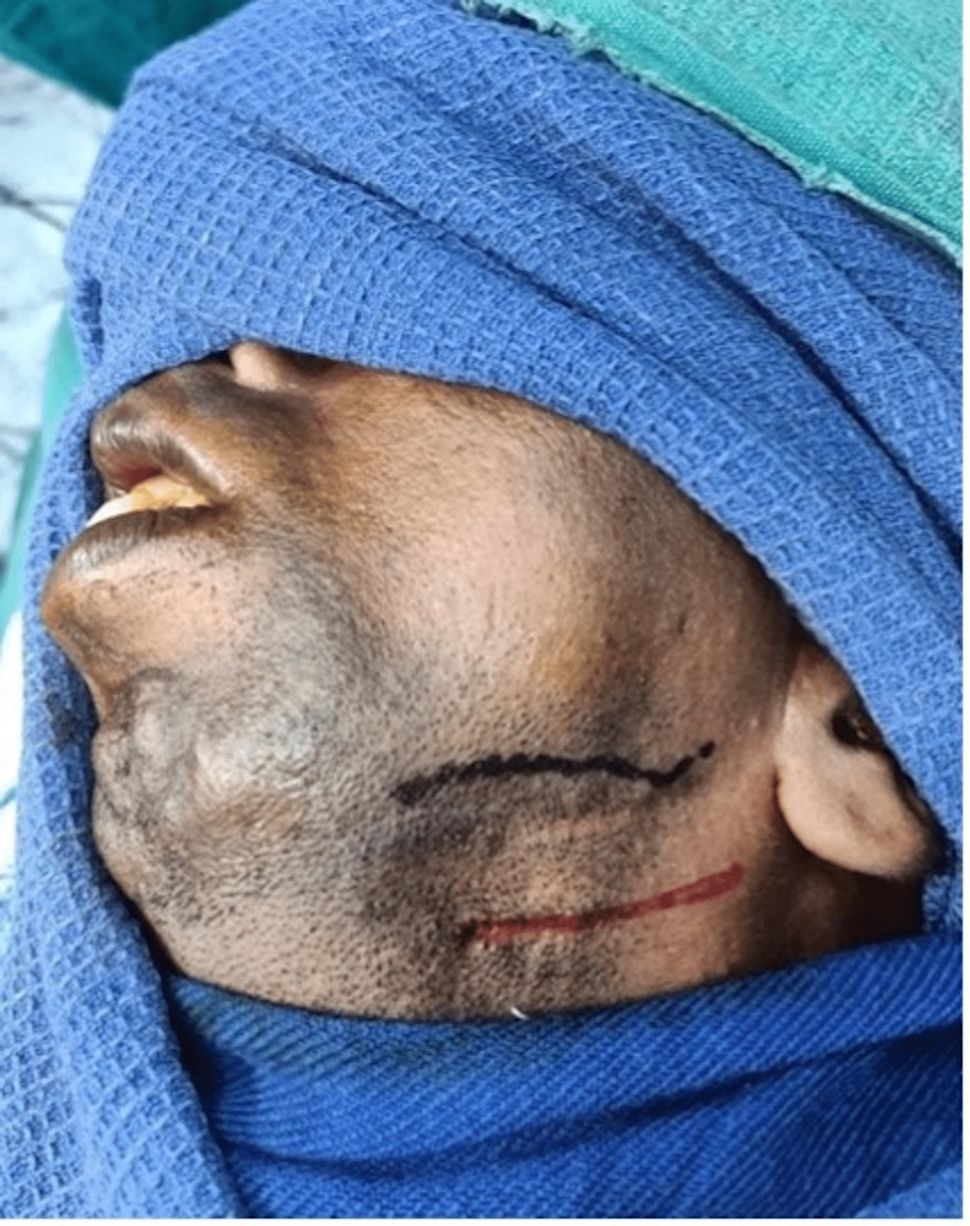
Marking of submandibular incision

**Figure 4:**
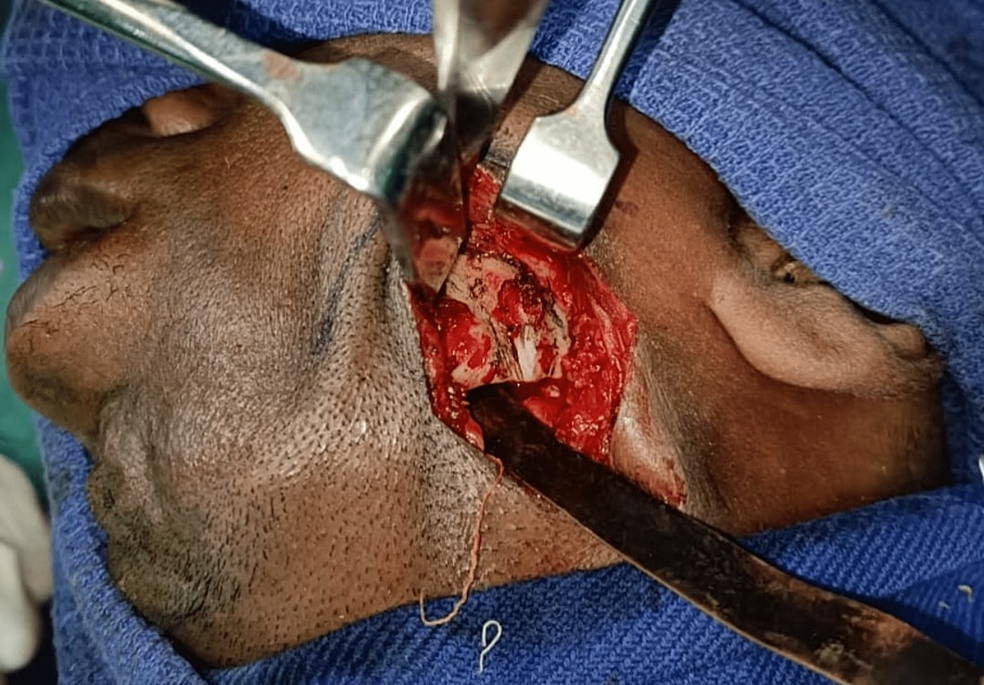
Marking of osteotomy site below the nerve canal

**Figure 5:**
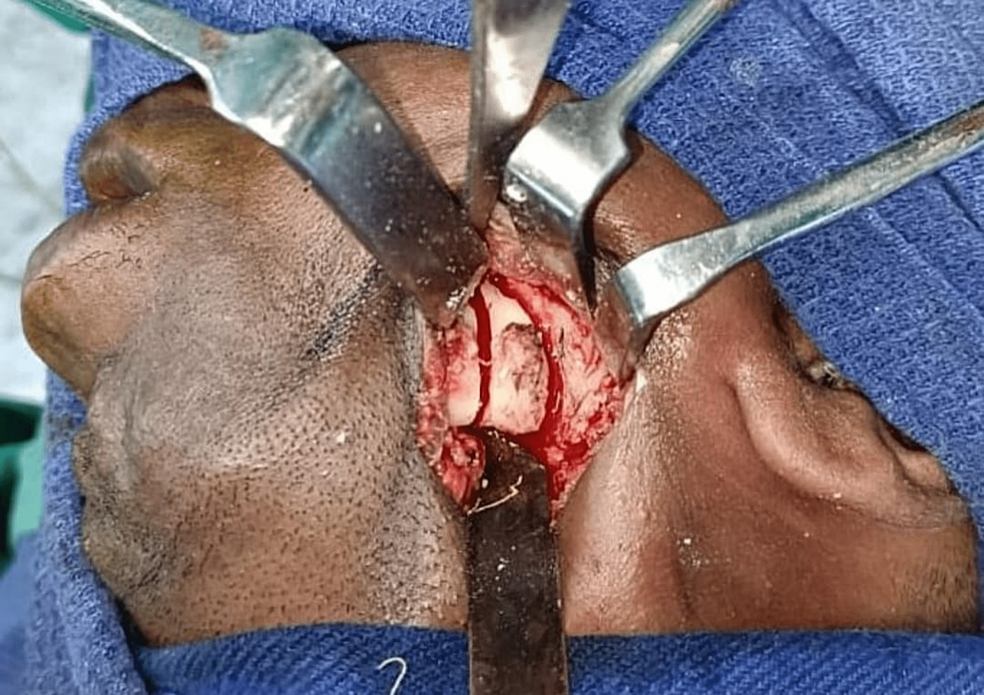
Osteotomy done below the nerve canal

**Figure 6:**
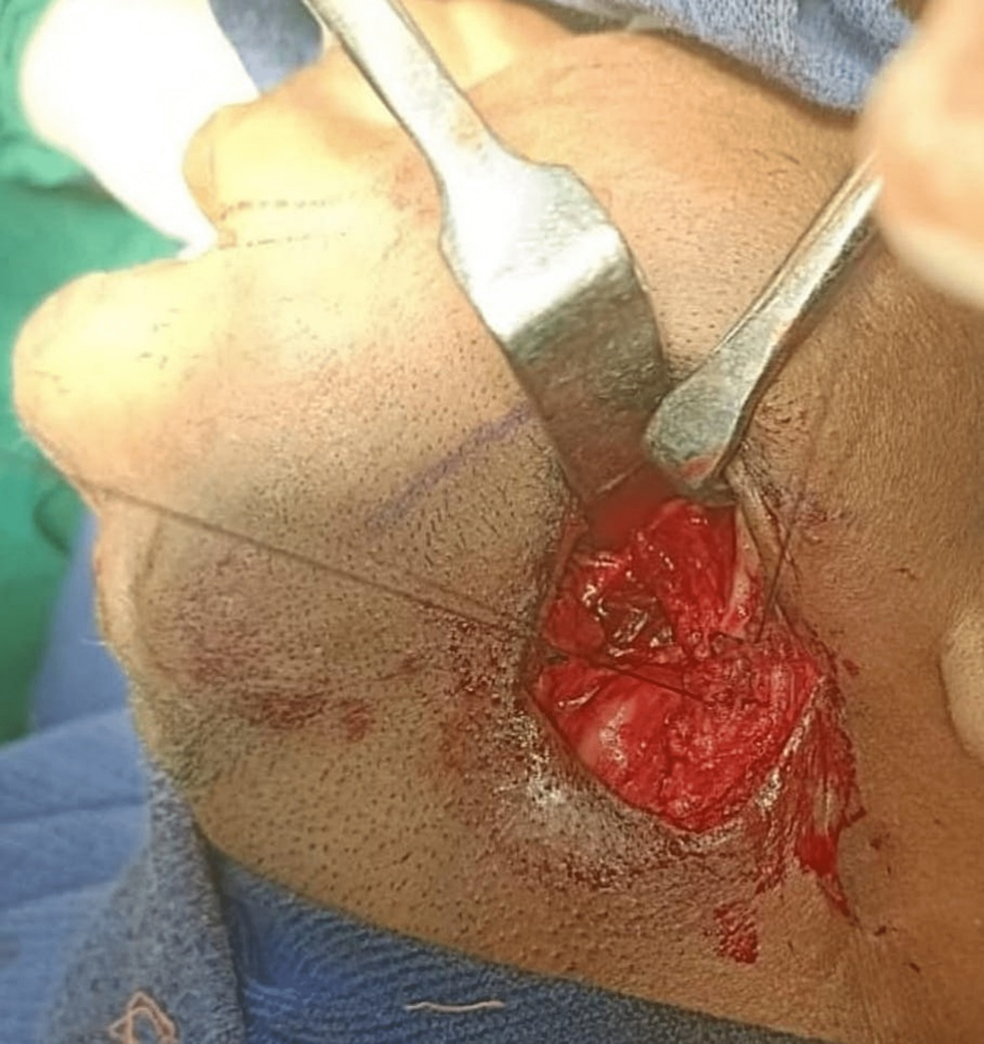
Interposition of the masseter and medial pterygoid between the osteotomy segments

**Figure 7:**
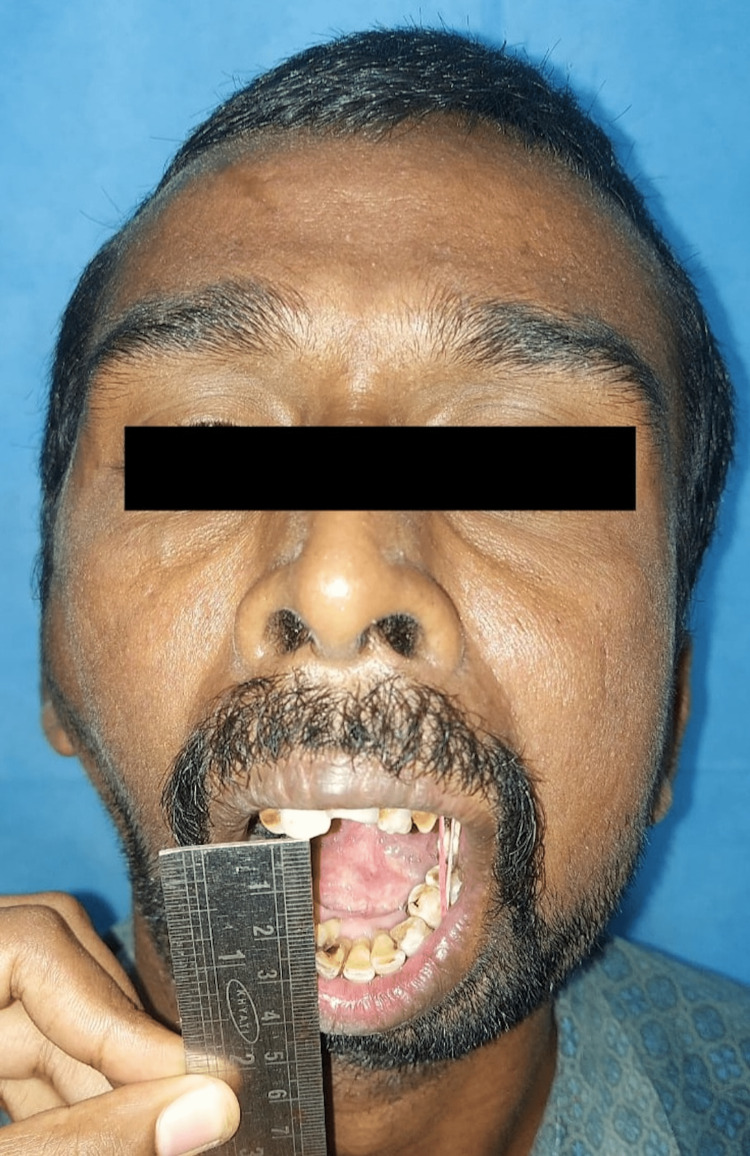
Postoperative mouth opening of 30 mm achieved

**Figure 8:**
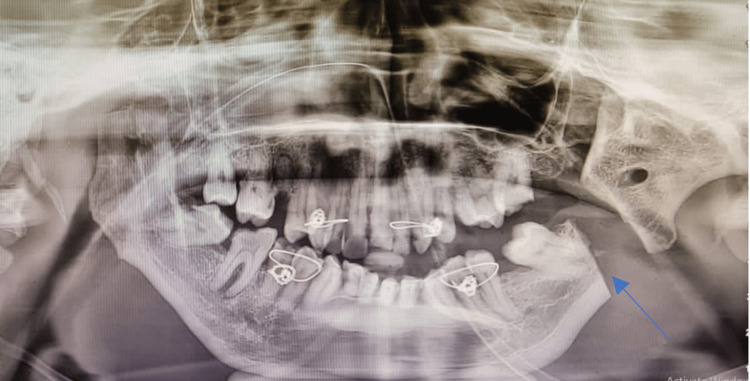
Postoperative orthopantomogram showing osteotomy done below the inferior alveolar nerve canal on the left side

**Figure 9:**
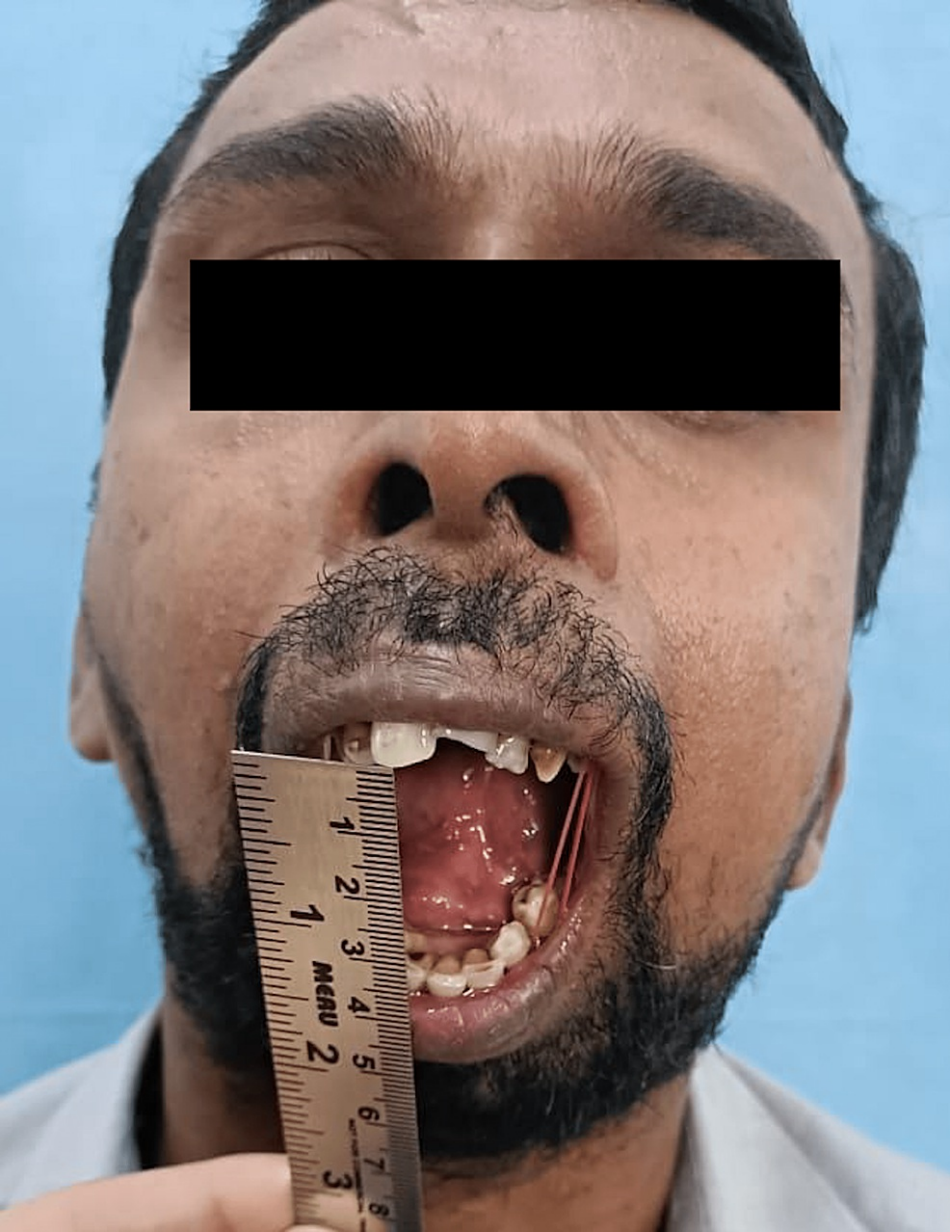
Mouth opening of 30 mm after one year

The body of the mandible was approached by a submandibular incision. Osteotomy sites were marked at the angle of the mandible, below the nerve canal, and approximately 2.5 cm bone was removed using 703 burs and osteotomes. Intraoperative hemorrhage was minimal. The medial pterygoid and masseter were interposed and sutured with 3-0 vicryl. This creates a pseudojoint, thus facilitating an improvement in mouth opening. The subcutaneous layer and skin were sutured with 3-0 vicryl and 4-0 prolene. Immediate postoperative mouth opening of 30 mm was achieved, and postoperative orthopantomogram shows osteotomy done at the angle region below the inferior alveolar nerve canal. Postoperative inferior alveolar nerve paresthesia was noted as the osteotomy was done below the nerve canal (Figures 3-9).

Modified Esmarch procedure

**Figure 10:**
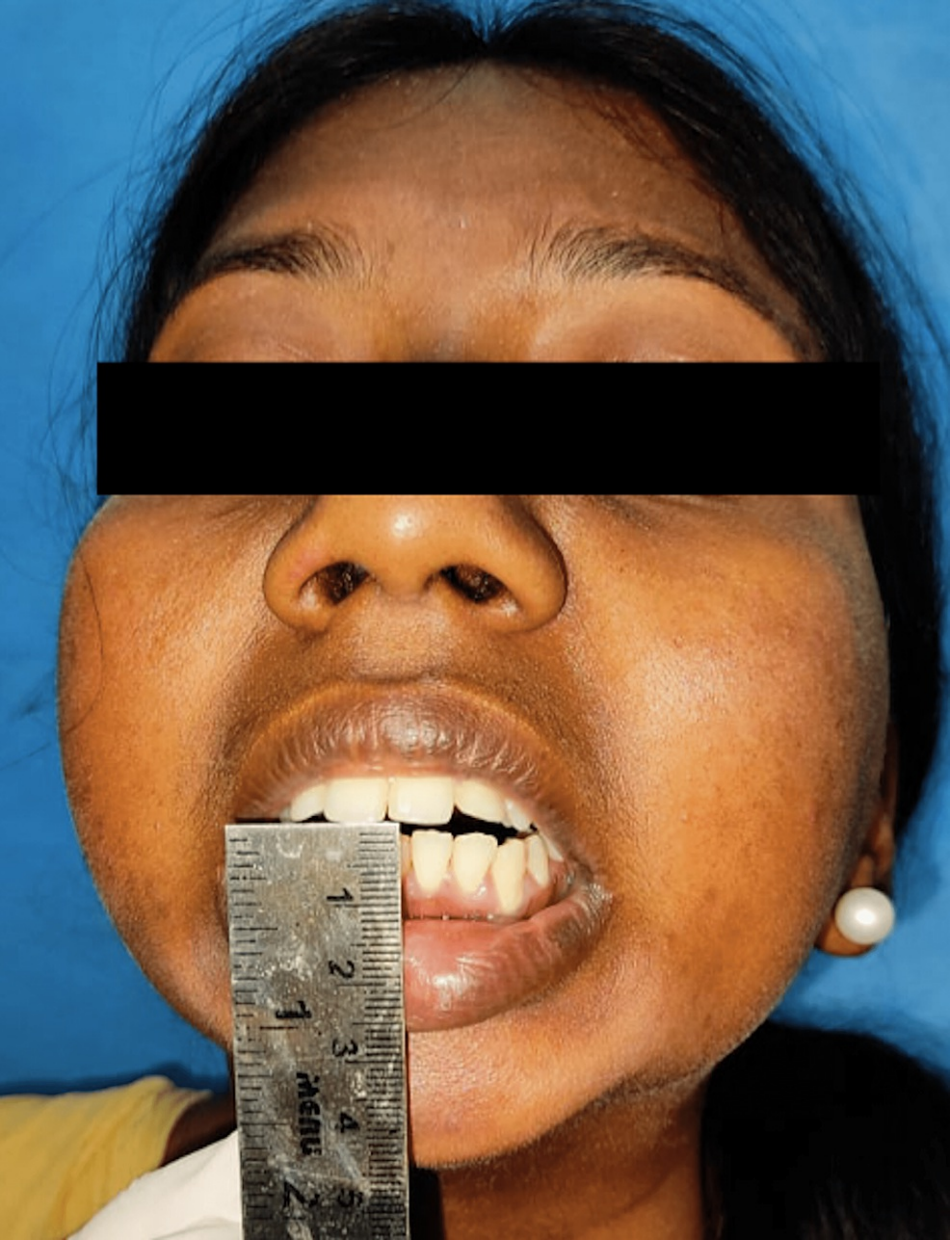
Preoperative nil mouth opening

**Figure 11:**
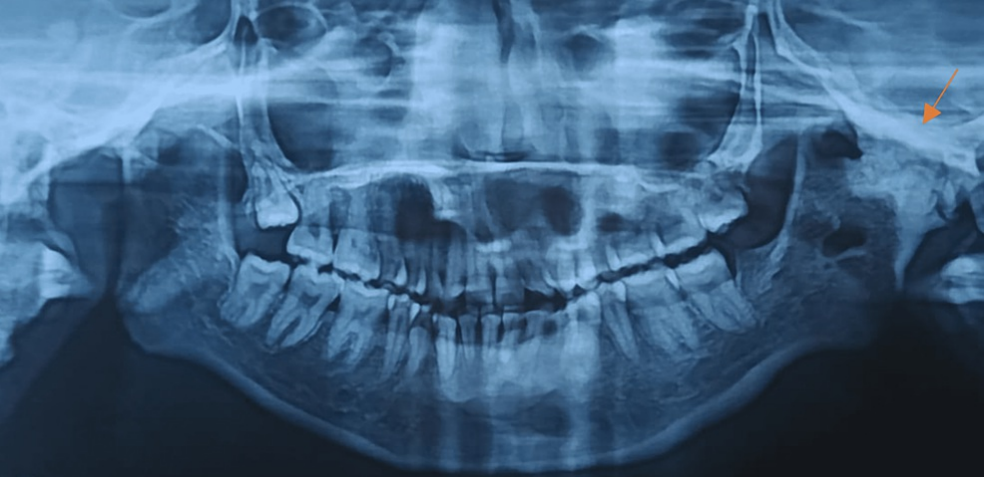
Preoperative orthopantomogram

**Figure 12:**
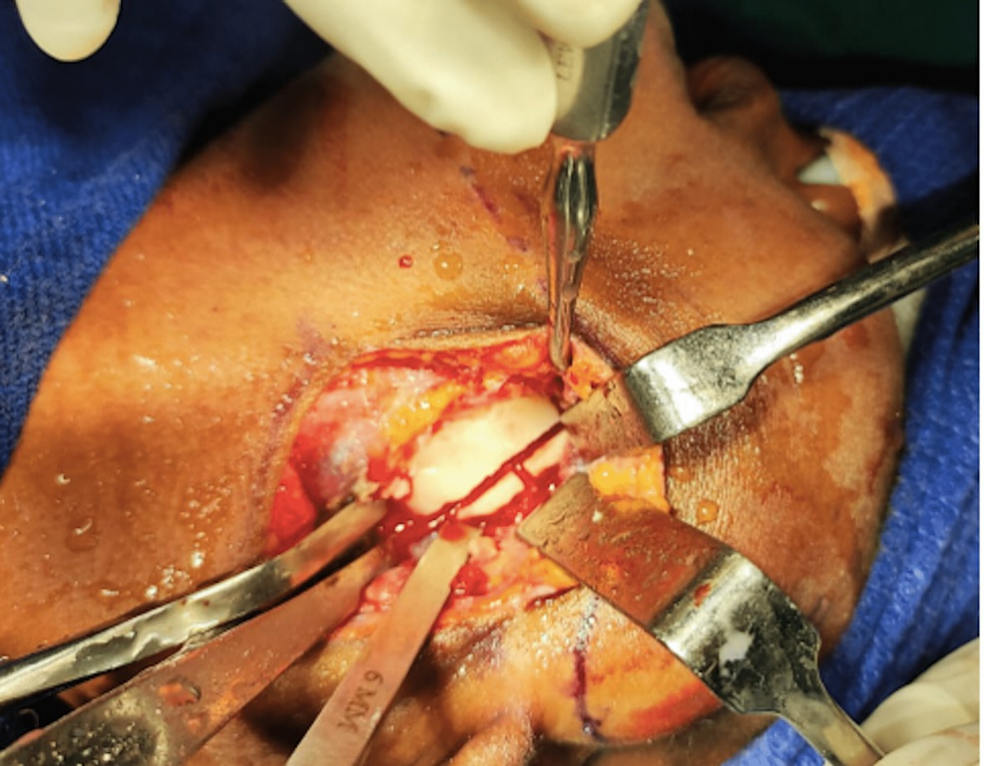
Modified osteotomy done above the inferior alveolar nerve canal

**Figure 13:**
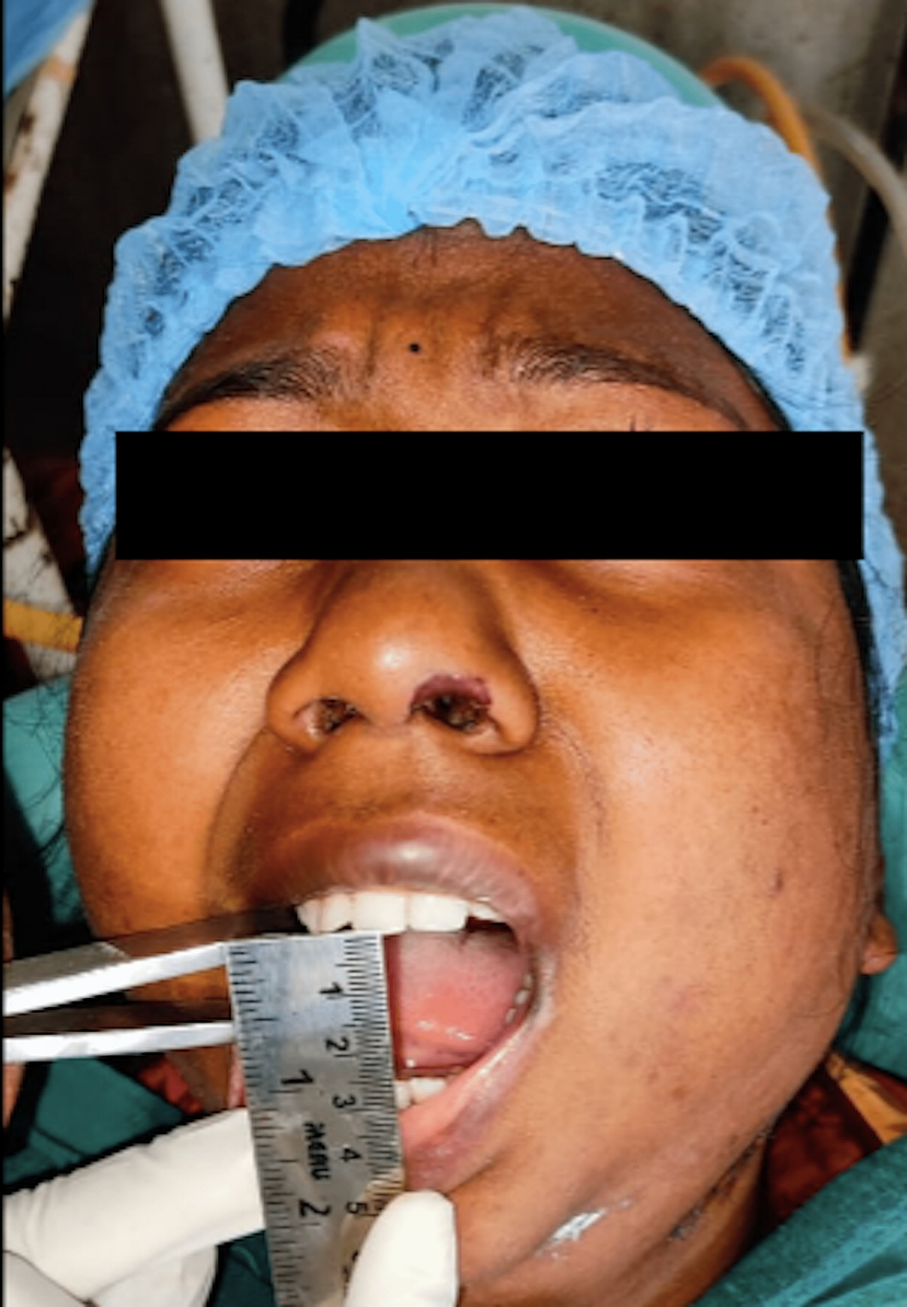
Postoperative mouth opening of 27 mm with physiotherapy

**Figure 14:**
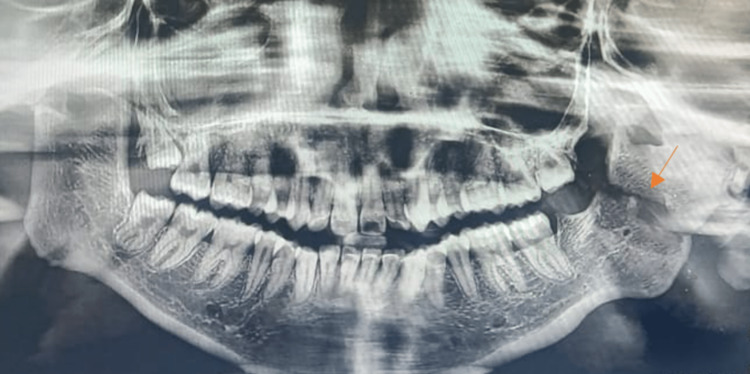
Postoperative orthopantomogram showing osteotomy done above the inferior alveolar nerve canal

**Figure 15:**
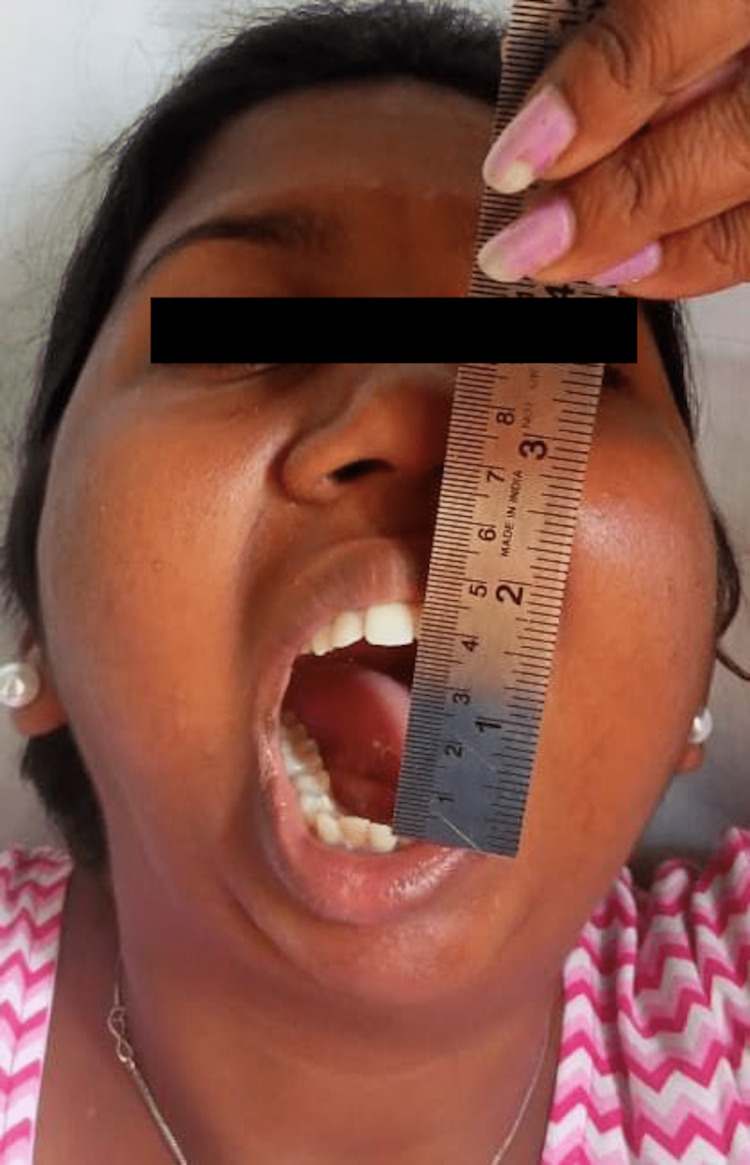
Mouth opening of 37 mm after one-year follow-up

In this case, as shown below, the patient encountered trauma to the jaws at the age of three years, following which there was a progressive decline in mouth opening (Figure 10). At 10 years of age, on the left side gap, arthroplasty was done, followed by interpositional arthroplasty with a rib graft three years later. Failure of aggressive physiotherapy postoperatively had led to reankylosis with nil mouth opening at the time of presentation. The current orthopantomogram revealed extensive callous formation on the left side (Figure 11). To avoid inferior alveolar nerve paresthesia, we decided to perform a modified Esmarch osteotomy, where the osteotomy cuts were made above the nerve canal (Figures 12-15 ). This caused a massive intraoperative hemorrhage due to the altered anatomy of the maxillary artery and was managed with the help of Flowseal.

The rest of the patients included in the case series presented with a similar history and clinical features and had undergone multiple surgeries for temporomandibular joint ankylosis. Owing to the altered vascular anatomy of the maxillary artery in reankylosis cases, to avoid massive intraoperative hemorrhage, in the remaining four cases, we decided to follow the conventional Esmarch procedure and placed the osteotomy cuts below the nerve canal. The patients were extubated on the next postoperative day, and a nasal airway was placed. Kazanjian buttons were placed postoperatively, and training elastics were placed. All patients were motivated to undergo aggressive postoperative physiotherapy. Postoperative mouth opening was measured with a scale in millimeters, and physiotherapy was advocated with the help of a heister to improve mouth opening.

## Results

**Figure 16:**
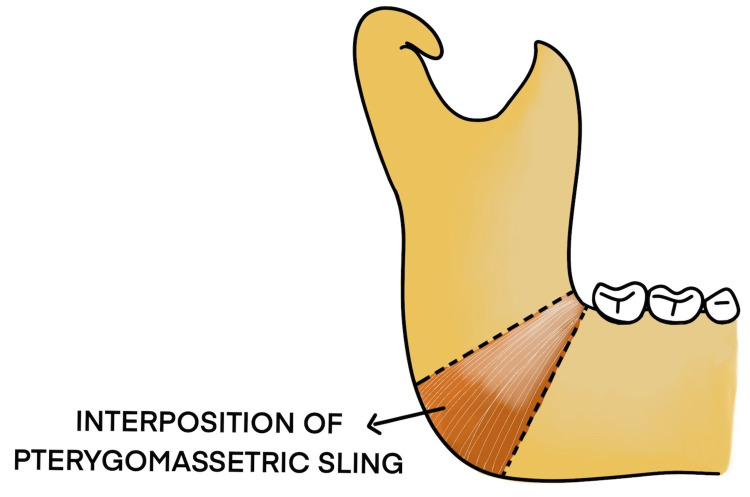
Interposition of pterygomassetric sling between the osteotomy segments

A total of six cases of temporomandibular joint reankylosis were operated using the Esmarch procedure. Five cases were operated with the conventional angle osteotomy and one case via the modified osteotomy. It was observed that in the modified Esmarch osteotomy, where the cuts were placed above the inferior alveolar nerve canal, there was a massive hemorrhage encountered intraoperatively. This was primarily attributed to the altered anatomy of the maxillary artery, which was very close to the ankylotic mass. This hemorrhage was managed with the help of a Flowseal placed at the surgical site. To avoid this intraoperative complication, we opted to perform the conventional Esmarch osteotomy for five patients, where the osteotomy was done below the inferior alveolar nerve canal. The pterygomassetric sling was then interposed between the osteotomy segments (Figure 16). It was found that by this conventional technique, the intraoperative hemorrhage was minimal, but it carried a risk of postoperative inferior alveolar nerve paresthesia.

Postoperative mouth opening achieved by this Esmarch procedure is shown in Table 1.

**Table 1:** Mouth opening assessment: preoperative, immediate postoperative, and after one-year follow-up in millimeters

Patient	Preoperative	Immediate postoperative	One-year follow-up
Patient 1	Nil	30 mm	31 mm
Patient 2	Nil	27 mm	37 mm
Patient 3	Nil	30 mm	32 mm
Patient 4	Nil	29 mm	34 mm
Patient 5	Nil	30 mm	36 mm
Patient 6	Nil	30 mm	32 mm

There are a few drawbacks associated with the Esmarch procedure, namely, inferior alveolar nerve paresthesia, deviation of mouth opening postoperatively, lack of balanced occlusion, and, since the osteotomy is in the ramus, distraction osteogenesis cannot be performed. Inferior alveolar nerve paresthesia can be avoided by placing the osteotomy above the nerve canal, but it carries a high risk for massive intraoperative hemorrhage from the maxillary artery. Five patients in whom conventional angle osteotomy was performed had postoperative inferior alveolar paresthesia, which was managed conservatively. There was no paresthesia postoperatively in one patient where the osteotomy was done above the nerve canal. Deviation of mouth opening and balanced occlusion were corrected with the help of training elastics. The immediate postoperative period is crucial due to the altered anatomy of the tongue and the pharyngeal muscles. Hence, swallowing exercises and retraining of tongue and circumoral musculature, along with elastics using Kazanjian buttons, improved the mandibular range of movements in a span of one week. The patients were motivated to do physiotherapy, and hence, postoperative mouth opening remained stable. All patients had satisfactory mouth opening in the immediate postoperative period and after the one-year follow-up period.

## Discussion

**Figure 17:**
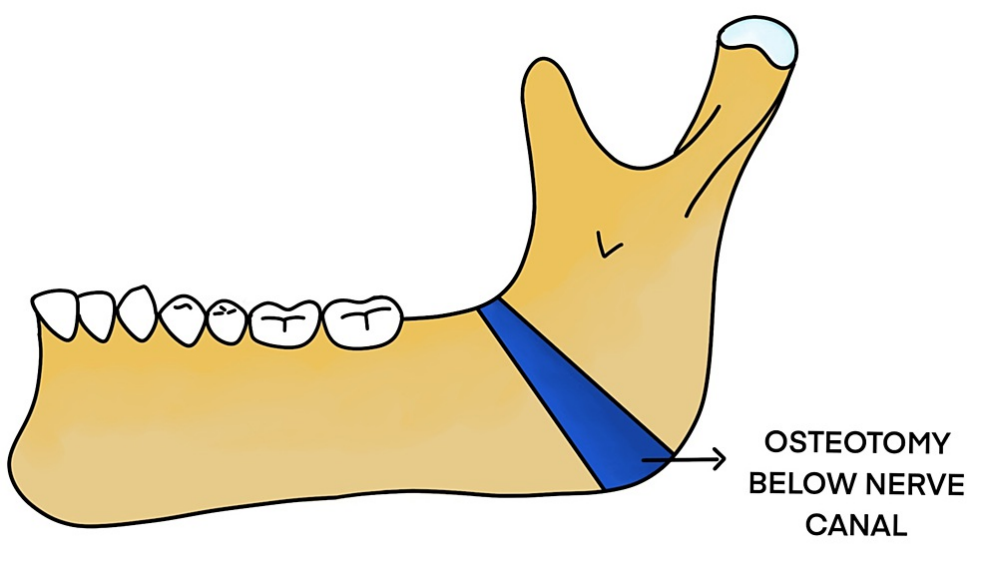
Esmarch angle osteotomy below the inferior alveolar nerve canal

**Figure 18:**
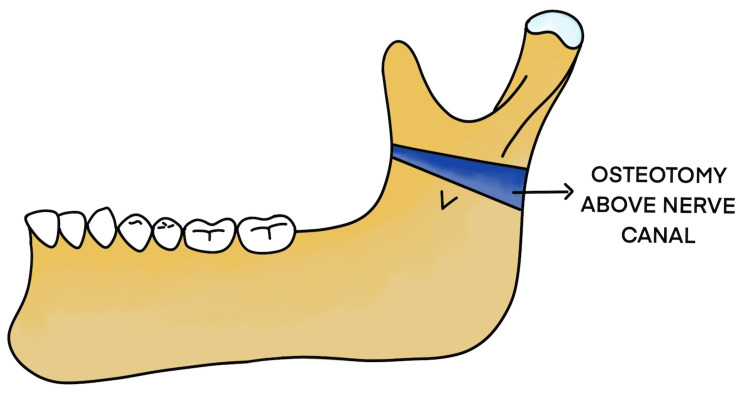
Modified osteotomy above the nerve canal

The treatment of temporomandibular joint ankylosis poses a significant challenge for the maxillofacial surgeon owing to its high incidence of reankylosis. Trauma is the most common cause for ankylosis to occur as stated by Kaban et al. [3]. The main goals of surgery are to achieve mouth opening and mandibular range of movements and improve the quality of life for these patients [4]. There are various surgeries for the release of ankylosis, dating back to the 19th century. Esmarch performed angle osteotomy to facilitate mouth opening by creating a pseudojoint in 1851 (Figures 17, 18).

**Figure 19:**
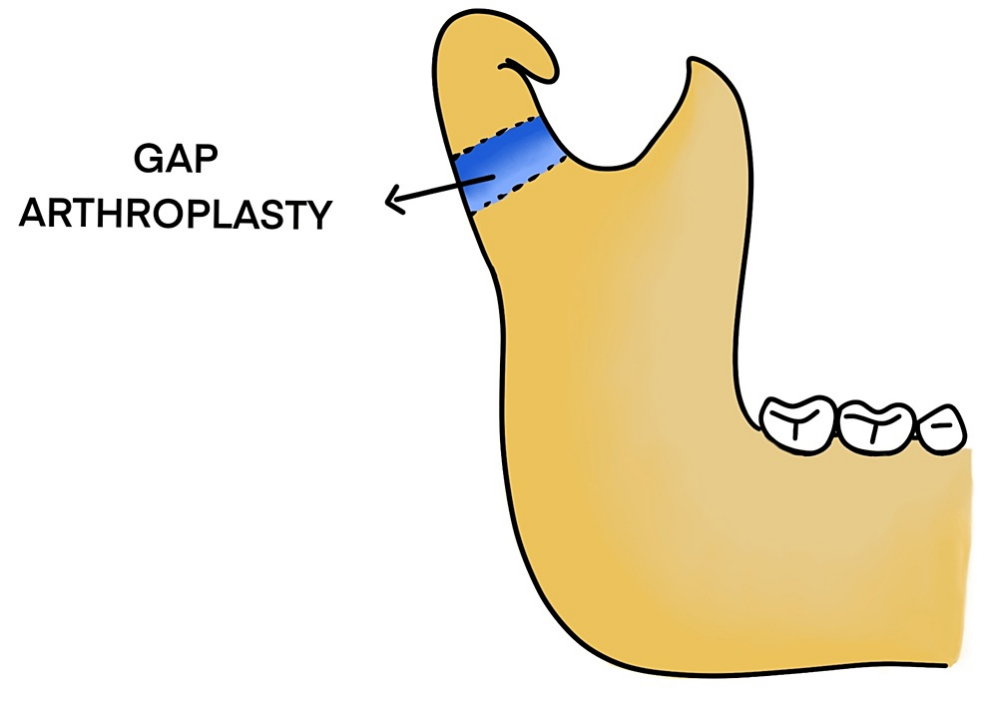
Gap arthroplasty

Other surgical methods include gap arthroplasty (Figure 19), interpositional arthroplasty, and transport distraction osteogenesis, with the recent development of total joint replacement prostheses [5].

Various interpositional materials used include pterygomassetric sling, temporalis myofascial flap, dermal fat graft, buccal fat pad graft, amniotic membrane, auricular cartilage, and costochondral graft [6]. Condylectomy was first advocated by Humphrey in 1826. Humphry analyzed temporomandibular joint ankylosis patients over a period of 10 years from 1850 to 1860, in which condylectomy and arthroplasty of the newly created joint cavity were performed using a myofascial flap [7]. Verneuil et al. in 1860 first used the temporalis muscle and fascia following gap arthroplasty. The first interpositional material used between the osteotomy segments was a pterygomassetric sling.

The conventional gap arthroplasty was popularized by Abbe [8], where there is complete resection of the ankylotic mass with no soft tissue present between the cut ends. Although it is less time-consuming, it has the drawbacks of causing ramal shortening, obstructive sleep apnea, reankylosis, and malocclusion [9]. Topazian [10] in 1966 showed that there was 53% more recurrence with gap arthroplasty when compared to interpositional arthroplasty. Salins [11] later modified the technique in 2000 by combining temporalis muscle and fascia with a block of autogenous cartilage or silastic about 6-8 mm thick while entirely omitting the creation of a gap arthroplasty. Clauser et al. [12] in their extensive studies showed that the main advantage of temporalis muscle and fascia flap was its resilience, autogenous nature, adequate blood supply, and its proximity to the joint, thus allowing it for a pedicled transfer into the joint area.

One of the most employed surgical techniques for temporomandibular joint ankylosis is gap arthroplasty, but it carries a risk of intraoperative hemorrhage. Studies have shown that the internal maxillary artery runs 3 mm medial to the mid-sigmoid notch [13]. Prasad et al. [14] in their study have analyzed the mean distance of the internal maxillary artery from the ankylotic mass. It was found to be on an average of 4 mm from the medial edge of the ankylotic mass. Schönegg et al. [15] have shown that extracranial hemorrhagic complications were most likely to arise from the maxillary artery, superficial temporal artery, and middle meningeal artery. The most commonly injured artery during the release of the ankylotic mass causing significant hemorrhage is the middle meningeal artery [15-17].

In a study by Talebzadeh et al. [18], it was shown that the middle meningeal artery lies 2.4 mm anterior from the height of the glenoid fossa. In one of our cases, due to the altered anatomy of the maxillary artery, there was a massive hemorrhage intraoperatively when the osteotomy cuts were placed above the nerve canal. Hence, for the remaining cases, osteotomy was done at the angle region, below the nerve canal.

In all our cases, the initial surgery performed via gap arthroplasty had a relapse, which led to reankylosis. In a study by Chen et al. [19], it was shown that the recurrence rate of reankylosis was 7.3%.

During the planning session with the radiologist, it was inferred that the ankylotic mass was extensive in one of our cases and was seen extending from the glenoid fossa to the condyle along with the involvement of the skull base and sphenoid and temporal bones. This creates inadequate space for joint movement, thus resulting in trismus. When there is extensive callous formation extending to the base of the skull, there is an increased risk of bleeding from the carotid arteries, due to skull base fracture, during the release of the ankylotic mass [20]. Hence, the Esmarch procedure was performed on one side and gap arthroplasty on the other side for this patient. There was minimal intraoperative hemorrhage when the osteotomy was done at the angle region, below the nerve canal.

The main drawback is the inadvertent inferior alveolar nerve paresthesia when the osteotomy is done below the inferior alveolar nerve canal. The incidence of postoperative inferior alveolar nerve paresthesia following various surgical procedures such as sagittal split osteotomy ranges from 9% to 84.6%, whereas it is 0.35%-8.4% in cases of impacted mandibular third molar removal [21,22]. Preoperatively, none of the patients had inferior alveolar nerve paresthesia. Postoperative inferior alveolar nerve paresthesia was observed in five patients with the conventional Esmarch procedure. In the process of creating a pseudojoint at the angle region, it was an untoward postoperative complication. There are various objective tests to assess neurosensory deficits such as mechanoreceptive or nociceptive tests. The treatment modalities include conservative methods and surgical repair. Initial conservative methods with corticosteroid therapy, pregabalin, and vitamin B12 supplements have been shown to reduce inflammation during the immediate postoperative period and can minimize the extent of nerve damage. In our cases, it was managed conservatively with tablet prednisone 30 mg tapered over a period of two weeks and tablet Neurobion Forte for the initial period of one month. The patients were under regular follow-up postoperatively. The patients noticed an improvement in altered sensation by the end of one month, and there was complete resolution after four months and by the end of one year during the postoperative follow-up period. According to the literature, several studies have shown successful resolution of inferior alveolar nerve paresthesia with conservative treatment methods [23]. Postoperative one-year follow-up of the patients showed no signs of paresthesia.

## Conclusions

Early intervention and aggressive postoperative physiotherapy along with retraining of circumoral and tongue muscles proved to be an important crucial factor to prevent reankylosis. Recent advances led to the development of total joint prosthesis, a promising technique in the management of temporomandibular joint ankylosis in adults.

In recurrent ankylosis cases, where there is extensive ankylotic mass extending from the glenoid fossa to the coronoid process of the mandible, this Esmarch procedure provides promising results when the osteotomy cuts are placed below the nerve canal. In five out of six cases, angle osteotomy was done below the nerve canal. This is a safer approach than the modified osteotomy but has the drawback of inadvertent inferior alveolar nerve paresthesia. Our experience with this Esmarch approach proves its versatility and efficacy and shines as a new thought of surgical approach with satisfactory mouth opening in temporomandibular joint reankylosis patients.
